# A novel member of the *let-7* microRNA family is associated with developmental transitions in filarial nematode parasites

**DOI:** 10.1186/s12864-015-1536-y

**Published:** 2015-04-22

**Authors:** Alan D Winter, Victoria Gillan, Kirsty Maitland, Richard D Emes, Brett Roberts, Gillian McCormack, William Weir, Anna V Protasio, Nancy Holroyd, Matthew Berriman, Collette Britton, Eileen Devaney

**Affiliations:** Institute of Biodiversity, Animal Health and Comparative Medicine, College of Medical, Veterinary and Life Sciences, University of Glasgow, Garscube Estate, Bearsden Road, Glasgow, G61 1QH UK; School of Veterinary Medicine and Science, University of Nottingham, Sutton Bonington Campus, Leicestershire, LE12 5RD UK; Advanced Data Analysis Centre, University of Nottingham, Nottingham, UK; Wellcome Trust Sanger Institute, Wellcome Trust Genome Campus, Hinxton, Cambridgeshire CB10 1SA UK

**Keywords:** *Brugia*, Lymphatic filariasis, Nematodes, microRNAs

## Abstract

**Background:**

Filarial nematodes are important pathogens in the tropics transmitted to humans via the bite of blood sucking arthropod vectors. The molecular mechanisms underpinning survival and differentiation of these parasites following transmission are poorly understood. microRNAs are small non-coding RNA molecules that regulate target mRNAs and we set out to investigate whether they play a role in the infection event.

**Results:**

microRNAs differentially expressed during the early post-infective stages of *Brugia pahangi* L3 were identified by microarray analysis. One of these, *bpa-miR-5364*, was selected for further study as it is upregulated ~12-fold at 24 hours post-infection, is specific to clade III nematodes, and is a novel member of the *let-7* family, which are known to have key developmental functions in the free-living nematode *Caenorhabditis elegans*. Predicted mRNA targets of *bpa-miR-5364* were identified using bioinformatics and comparative genomics approaches that relied on the conservation of *miR-5364* binding sites in the orthologous mRNAs of other filarial nematodes. Finally, we confirmed the interaction between *bpa-miR-5364* and three of its predicted targets using a dual luciferase assay.

**Conclusions:**

These data provide new insight into the molecular mechanisms underpinning the transmission of third stage larvae of filarial nematodes from vector to mammal. This study is the first to identify parasitic nematode mRNAs that are verified targets of specific microRNAs and demonstrates that post-transcriptional control of gene expression via stage-specific expression of microRNAs may be important in the success of filarial infection.

**Electronic supplementary material:**

The online version of this article (doi:10.1186/s12864-015-1536-y) contains supplementary material, which is available to authorized users.

## Background

The molecular mechanisms by which eukaryotic pathogens infect their hosts remain key questions in infection biology, as improved understanding of these events could lead to new methods of control. This is particularly so for vector-borne parasites, where the ability to survive and differentiate within the new environment of a mammalian host is critical for successful transmission. In this paper we explore the molecular events underlying the transmission of filarial nematodes from the mosquito to the mammalian host. We focus on microRNAs (miRNAs), which are small (~22 nucleotide) non-coding RNAs that regulate gene expression in animals, plants and viruses. miRNAs bind to complementary sequences, often in the 3′ untranslated region (UTR) of mRNAs, leading to translational repression and mRNA destabilisation [1]. miRNAs are essential during development in the free-living nematode *Caenorhabditis elegans,* with functions in developmental timing [2-4], lifespan and stress responses [5,6], and embryogenesis [7,8]. *C. elegans lin-4*, the first miRNA to be identified [2], regulates developmental switching during the early larval stages (L1 and L2) by repressing the heterochronic proteins LIN-14 [9] and LIN-28 [10], allowing epidermal cell fate transitions. *C. elegans let-7* was the second miRNA to be identified and acts from the L3 stage onwards to regulate the transition from L4 to adult by down-regulating a number of targets, including LIN-41 [3,11]*. let-7* is the founding member of a family of *C. elegans* miRNAs that share identity in the seed sequence (5′ nucleotides 2–7). This family includes a further six miRNAs; *miR-48, miR-84, miR-241, miR-793, miR-794,* and *miR-795* [12]. While *let-7* is involved in specifying larval-adult cell fate, *miR-48*, *miR-84* and *miR-241* are co-activated at an earlier time point where they co-ordinate the L2-L3 transition [4].

Although information continues to accrue on miRNAs in the model nematode (reviewed recently in [13]), much less is known in parasitic nematodes. We recently identified 104 miRNAs from the filarial nematode *Brugia pahangi* [14], the sister species to the human pathogen *B. malayi*. In our study the miRNA identification process required that all sequences matched perfectly to the *B. malayi* genome and we therefore consider these miRNAs common to both species, with a recent study confirming this [15]. *B. malayi* and the related *Wuchereria bancrofti* are the causative agents of lymphatic filariasis. Collectively, filarial worms infect around 120 million individuals in 73 countries worldwide [16,17]. These parasites cause a wide spectrum of pathology in the infected individual, including the debilitating conditions of elephantiasis and hydrocele. Lymphatic filariae have complex life cycles with no free-living forms; the parasites develop from first-stage larvae (microfilariae) to infective L3 within the mosquito intermediate host, and then from L3 to adults within the mammalian definitive host. Following transmission from an infected mosquito, the L3 migrate to the lymphatics where they develop through two moults to adults, which have a reproductive life-span of approximately 8 years [18]. Mated females release millions of microfilariae into the bloodstream where they are available for ingestion by a mosquito taking a blood meal. Lymphatic filariae therefore have two periods of developmental arrest, as L3 in the mosquito and as microfilariae in the blood of the mammalian host. While parasite development is dependent upon transmission between hosts, little is known of the molecular mechanisms that control arrest and development within the different hosts.

In this paper we show that a novel member of the *let-7* miRNA family, *bpa-miR-5364*, is highly up-regulated to coincide with the transition of the L3 from mosquito to mammalian host. The role of *bpa-miR-5364* in the infection event was investigated by identifying potential mRNA targets using bioinformatic predictions, comparative genomics, and transcriptomic analysis, with selected targets verified using a mammalian cell transfection system. We propose that by regulating specific mRNAs, *bpa-miR-5364* plays a key role in the transmission of the L3 stage from mosquito to mammalian host. This is the first example of a parasitic nematode miRNA for which function has been investigated and its mRNA targets identified and confirmed experimentally.

## Results

### Expression profiling identifies developmentally regulated *B. pahangi* miRNAs

The complete lifecycle of *B. pahangi* is maintained in our laboratory allowing access to specific developmental stages. Therefore, from the miRNAs found in our previous genome-wide discovery study [14], we aimed to identify those with important developmental functions by detailed expression profiling at six key developmental time-points. The following life cycle stages were analysed: mosquito-derived L3, L3 isolated from the mammalian host at day 1 and day 5 post-infection (p.i.), L4 isolated at day 10 p.i., and sexually mature adult males and females isolated approximately 3 months p.i. The mosquito-derived L3 and day 1 p.i. L3 time-points were selected in order to identify changes occurring coincident with infection, day 5 p.i. L3 and day 10 p.i. L4 to identify changes prior to and after a moult, and males and females to define miRNA profiles specific to the adult stages. RNA was prepared from worms recovered at each time-point using six biological replicates, with three replicates used for microarray and three for qRT-PCR. A custom array (LC Sciences) contained probes against *Brugia* spp. [14,19] and *C. elegans* (miRBase release 15) miRNA sequences. This paper focuses on miRNAs differentially expressed in the infective stage as it transits from the mosquito to the mammalian host; our analysis of miRNAs functioning specifically in adult parasites will be presented elsewhere. The microarray data for the larval stages is provided in Additional file 1.

### Significant changes in miRNA profiles are linked to invasion of the host

**Figure 1:**
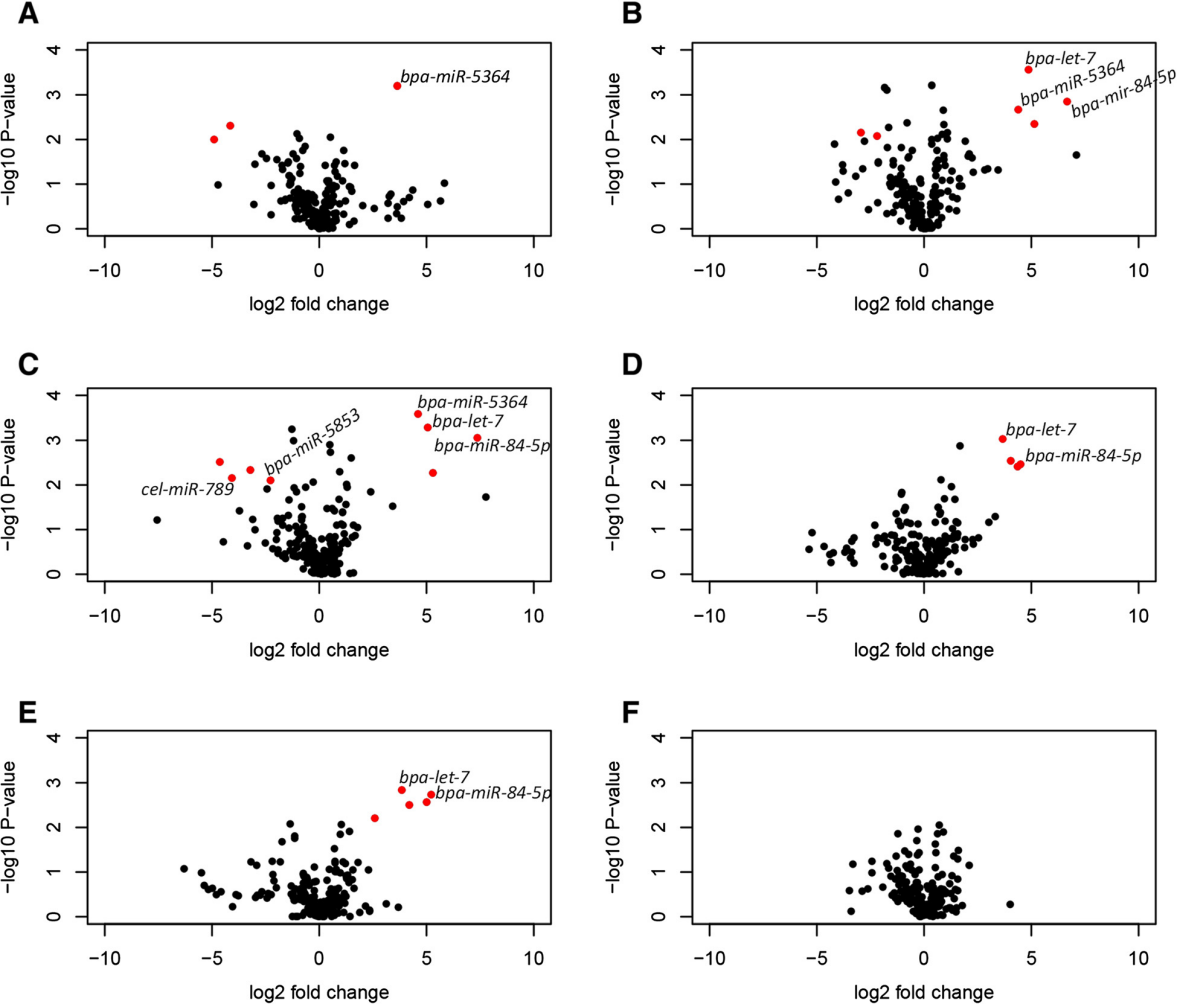
*Brugia* miRNAs are developmentally regulated. Volcano plots of pairwise comparison for larval stage microarray results. Probes in red represent those with *t*-test p-values of <0.01 (log_10_ 1.9) and Log_2_ fold changes of >2. **A)** mosquito-derived L3 versus day 1 p.i. L3, **B)** mosquito-derived L3 versus day 5 p.i. L3, **C)** mosquito-derived L3 versus day 10 p.i. L4, **D)** day 1 p.i. L3 versus day 5 p.i. L3, **E)** day 1 p.i. L3 versus day 10 p.i. L4, **F)** day 5 p.i. L3 vs day 10 p.i. L4.

To identify high confidence sets of miRNAs that were differentially expressed during larval development, the processed microarray results were filtered to retain only those with *t*-test *p*-values of < 0.01 and Log_2_ fold changes of > 2 between two larval time-points (Figure 1 and Additional file 2). These criteria identified a set of 13 probes which were then further refined to five differentially expressed miRNAs (as described in the legend for Table 1). Importantly, this set included the *let-7* miRNA family members *bpa-let-7* and *bpa-miR-84*, suggesting that other *B. pahangi* miRNAs with important developmental functions could potentially be identified by this analysis.

**Table 1:** **Mean** ***bpa-miR-5364*** **microarray signal in five** ***B. pahangi*** **life cycle stages**

**Reporter name** ^**(a)**^	**Mosquito-derived L3**	**Day 1**	**Day 5**	**Day 10**	**Adult male**	**Adult female**
*bpa-let-7*	93	214	2,713	3,068	2,678	1,398
*bpa-miR-84-5p*	4	18	409	669	1,134	758
*bpa-miR-5364*	708	8,794	14,922	17,301	9,173	8,518
*cel-miR-789*	808	2,165	58	48	213	26
*bpa-miR-5853*	1,329	2,079	636	275	933	207

^(a)^Eight probes shown in Additional file 2 are not included here for the following reasons: *cel-let-7*, as its profile was essentially identical to *bpa-let-7*; bpa-miR-84-3p*, as it sequence is identical to *bpa-miR-84-5p*; Bpa0107, as its sequence corresponds to a tRNA [14]; bpa-miR-5853*, cel-miR-74 and *bpa-miR-5872*, due to low expression (defined as signal <500 for all replicates of the two time-points where expression was significantly different); *cel-mir-51* and *cel-mir-266*, as the corresponding *B. pahangi* sequences could not be identified by PCR amplification of candidate sequences found from re-analysis of our previous small RNA deep sequence data [14].

In addition to *bpa-let-7* and *bpa-miR-84,* the three other miRNAs that met the criteria were *bpa-miR-5364, cel-miR-789 and bpa-miR-5853*. We chose to focus on *bpa-miR-5364* for the following reasons: its expression profile is particularly striking as it is up-regulated ~12 fold (Log_2_ 3.64) within 24 hours of infection of the mammalian host and doubles again over the period to day 10 p.i. (Table 1); *bpa-miR-5364* shares sequence similarity with the *let-7* miRNA family and appears to be specific to clade III parasitic nematodes (as described below); it is one of the most abundant miRNAs with only *bpa-lin-4* and *bpa-miR-71* found at higher levels at day 10 p.i. (Additional file 1). Abundant miRNA are often evolutionarily ancient [20] suggesting important function.

### *bpa-miR-5364* is a novel member of the *let-7* miRNA family

**Figure 2:**
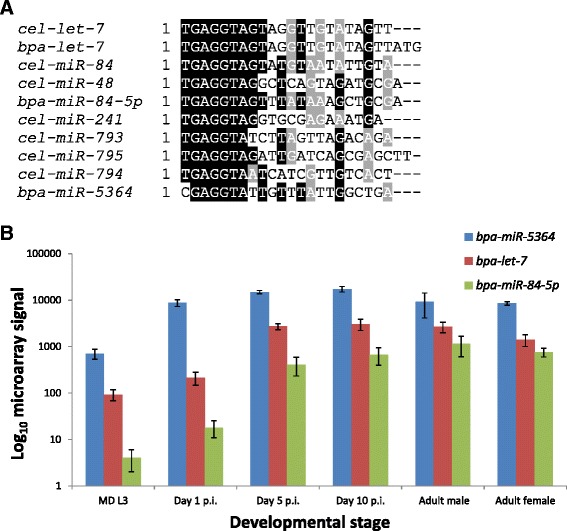
*miR-5364* is a novel member of the *let-7* miRNA family. **A**. CLUSTAL alignment comparing miRNA sequences from the *C. elegans let-7* family with *bpa-miR-5364, bpa-let-7* and *bpa-miR-84*. Shaded nucleotides are identical. Note the level of identity particularly over the seed sequence (5′ nucleotides 2–7). **B**. Abundance of *bpa-miR-5364*, *bpa-let-7* and *bpa-miR-84* increase in parallel from mosquito-derived (MD) L3 to adult as determined by microarray analysis (log_10_ scale). The mean of three biological replicates is shown +/− standard deviation.

In the free-living nematode *C. elegans*, the *let-7* miRNA family controls developmental transitions and consists of seven members of which only two (*let-7* and *miR-84*) are found in *Brugia* spp. [14,15,19]. Although *bpa-miR-5364* is clearly a novel sequence, it can be considered a member of the *let-7* family due to conservation around the miRNA seed sequence (Figure 2A). This led us to hypothesise that *bpa-miR-5364* may also influence a developmental transition, in this case from the mosquito vector to the mammalian host. Comparison of the overall microarray expression profiles of *bpa-miR-5364* with the *B. pahangi let-7* family miRNAs reveals a related but distinct pattern: while the expression profiles of all three miRNAs mirror each other, *bpa-miR-5364* is found at a much higher level than *bpa-let-7* and *bpa-miR-84* and is up-regulated a minimum of 24 hours earlier (Figure 2B).

### Expression of *bpa-miR-5364* is induced in culture

**Figure 3:**
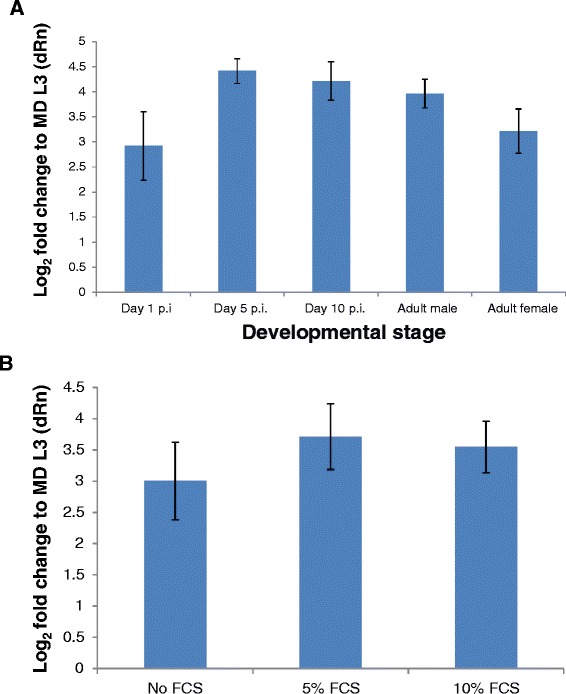
Expression of *bpa-miR-5364* is upregulated following host invasion and in cultured larvae. **A**. qRT-PCR confirms *bpa-miR-5364* is up-regulated between mosquito-derived L3 and 24 h p.i. L3. Levels of *bpa-miR-5364* were assessed relative to the constitutively expressed *bpa-mir-100c* and are expressed as log_2_fold change relative to mosquito-derived (MD) L3*.* The mean of three biological replicates is shown +/− standard deviation; replicates were independent from those used in the microarray analysis. **B**. *bpa-miR-5364* expression determined by qRT-PCR in *B. pahangi* L3 cultured at 37°C in worm culture medium that was either FCS-free, or contained 5% or 10% FCS. Levels of *bpa-miR-5364* were normalised to *bpa-miR-100c* and are expressed as log_2_fold change relative to mosquito-derived (MD) L3. The mean of three technical replicates from a single biological replicate is shown +/− standard deviation.

The *bpa-miR-5364* developmental expression profile defined by microarray was confirmed by qRT-PCR, with the striking increase in abundance of the mature miRNA clearly evident within 24 hours post-infection of the mammalian host (Figure 3A). To test if this rapid increase in *bpa-miR-5364* level could be replicated *in vitro*, mosquito-derived L3 were cultured for 24 hours at 37°C in medium without serum, or with the addition of 5% or 10% serum. qRT-PCR demonstrated that *bpa-miR-5364* abundance increased after 24 hours in culture to a level similar to that found in 24 hour p.i. L3 analysed directly *ex vivo*, with the addition of serum causing no significant difference (P > 0.2 for both concentrations of FCS vs no FCS, unpaired *t*-test) (Figure 3B).

To examine potential mechanisms regulating expression, the genomic sequences 5′ and 3′ of *bpa-mir-5364* were analysed. *Brugia* spp. *mir-5364* is an intergenic miRNA with the closest protein coding region residing 1.2 kb upstream and is therefore likely to be regulated by its own promoter. However, analysis of this region revealed no significant transcription factor binding sites (not shown). In *C. elegans, let-7* is autoregulated via a *let-7* binding site situated at the 3′ end of the primary transcript approximately 500 bp downstream of the sequence encoding the mature miRNA [21]. Autoregulation of *C. elegans lin-4* also occurs, in this case by activation of *lin-4* transcription via a *lin-4* binding site in its promoter [22]. The *B. malayi mir-5364* genomic sequence was analysed using the Probability of Interaction by Target Accessibility (PITA) algorithm [23]. Interestingly, predicted binding sites for *mir-5364* were found 614 bases downstream (7 nucleotide seed containing one G:U pairing, ΔΔG of −6.15) and 798 bases upstream (8 nucleotide seed containing one G:U pairing, ΔΔG −11.21) of the *mir-5364* mature region. This suggests that similar mechanisms of autoregulation to those described for *let-7* and *lin-4* in *C. elegans* might exist for *mir-5364* in the parasite.

### *mir-5364* is specific to clade III parasitic nematodes

miRBase (release 21) (http://www.mirbase.org/) shows that, to date, *mir-5364* has been identified only in the parasitic nematodes *B. pahangi* [14] and *Ascaris suum* [24], both of which are members of nematode clade III [25]. *mir-5364* is not present in the deep-sequencing-defined miRNA sets from the animal parasitic nematodes *Haemonchus contortus* (clade V) [14] or *Strongyloides ratti* (clade IVa) [26], nor in the free-living nematode species *C. elegans, C. briggsae, C. brenneri, C. remanei, Pristionchus pacificus* (all clade V) or *Panagrellus redivivus* (clade IV) [26-29], suggesting that *mir-5364* miRNA is specific to clade III parasitic nematodes. To investigate this further, we examined a number of other nematode genomes to determine the extent of *mir-5364* conservation. Using the *bpa-mir-5364* precursor sequence as a query for BLASTN analysis at 959 Nematode Genomes (http://xyala.cap.ed.ac.uk/downloads/959nematodegenomes/blast/blast.php, with default settings), *mir-5364* was found in all clade III parasitic nematode genomes for which sequence was available. Regions of ≥ 84% identity were identified in the filarial nematodes *Dirofilaria immitis, Loa loa, Wuchereria bancrofti*, *Litomosoides sigmodontis* and *Onchocerca ochengi* (identity ranging from 66/78 nucleotides in *O. ochengi* to 128/137 nucleotides in *W. bancrofti*) with complete conservation of the mature miRNA sequence in all five species (Additional file 3). When folded using mfold [30] (http://mfold.rit.albany.edu/?q=mfold/RNA-Folding-Form, default settings) the regions of precursor homology identified from each species formed miRNA-like hairpins (Additional file 3) which also passed the miRNA hairpin classifier algorithm miPred (http://www.bioinf.seu.edu.cn/miRNA/index.html) [31] (Additional file 4). In contrast, our bioinformatic analyses showed no evidence for *mir-5364* orthologs in the genomic data at 959 Nematode Genomes of the non-clade III nematodes *Bursaphelenchus xylophilus, Heligosomoides polygyrus, Howardula aoronymphium* or *Oscheius tipulae* using either the precursor or mature sequences as BLAST queries. Although it is possible that divergent sequences might not have been detected using BLASTN, *miR-5364* was not found in *H. polygyrus* by deep sequencing of small RNAs [32]. Therefore, as *miR-5364* was also recently experimentally confirmed in *B. malayi* [15], this miRNA is highly conserved in eight clade III parasitic nematodes but has not been identified in any other species to date.

### Computational and phylogenetic analysis predicts target mRNAs

As miRNAs bind to partially complementary sequences in their target mRNAs, computational analysis can be used to predict targets [33,34]. Genome-wide target predictions for *miR-5364* were performed utilising *B. malayi* and *D. immitis* genomic data [35,36]. Although miRNA target sites have been defined throughout genes [37], we focussed on the 3′UTR, as many well-characterised target sites reside in this region [1,9,11,38,39]. Also, rather than rely on annotated 3′UTRs, which are often dependent on developmental stage [40] or cell type, and for which the current *B. malayi* and *D. immitis* data are incomplete, we used 1 kb of genomic sequence downstream of the annotated end of all genes. The miRNA target prediction algorithms PITA [23] and miRanda [41] were used and only predictions found using both programs were retained. The genome-wide *miR-5364* target predictions for both parasites are provided in Additional file 5.

To identify high confidence *miR-5364* targets, the large number of predictions were refined by first considering only those with PITA ΔΔG energies of < −7. This resulted in 923 target sites in 857 *B. malayi* genes and 948 targets in 858 *D. immitis* genes. We then defined the genome-wide set of *B. malayi* and *D. immitis* orthologs using InParanoid [42] (Additional file 6) and retained only those genes where a *miR-5364* binding site was found in the downstream region of orthologous genes. This resulted in 75 predicted *miR-5364* binding sites in 68 *B. malayi* genes, and 72 sites in 64 *D. immitis* genes (Additional file 6). Target-site positioning was then considered and predictions retained only if the site occurred with similar positioning (as defined in Materials and Methods) in the two species. This process produced a final set of 13 orthologous genes that were high confidence predicted targets of *miR-5364* (Table 2 and Additional file 6). Three of these genes were chosen for further study; Bm1_27305 (encoding an ETS-domain containing protein), Bm1_05425 (encoding a Zinc finger in N-recognin family protein) and Bm1_25620 (which encodes a putative high mobility group, HMG, protein). For Bm1_27305 and Bm1_05425 the *miR-5364* target sites were <100 nucleotides downstream of the stop codon, an arrangement that can result in potent silencing [43], and the target site positioning was within one nucleotide and 11 nucleotides, respectively, in the two species (Additional file 6). The third predicted target, Bm1_25620 was included as proteins of this type are known DNA-binding proteins [44]. Further support that our predictions represented *bona fide miR-5364* targets was provided by searching for sites in the orthologs of Bm1_27305, Bm1_05425 and Bm1_25620 in the clade III parasitic nematodes *L. loa*, *W. bancrofti* and *L. sigmodontis* (see Additional file 7). 3′ RACE was performed for the three proposed target genes, using *B. pahangi* material, which confirmed the annotated stop and the presence of the *miR-5364* target sequence in the 3′UTRs.

**Table 2:** **Predicted mRNA targets of** ***bpa-miR-5364***

***B. malayi g*** **ene annotation**	***B. malayi*** **ortholog**	***D. immitis*** **ortholog**	***C. elegans*** **ortholog** ^**(a)**^
Delta-1-pyrroline-5-carboxylate dehydrogenase, mitochondrial precursor, putative	Bm1_04205	nDi.2.2.2.g07494	F56D12.1a
Zinc finger in N-recognin family protein	Bm1_05425	nDi.2.2.2.g05278	T22C1.1
Twik (KCNK-like) family of potassium channels, alpha subunit 5	Bm1_15670	nDi.2.2.2.g02778	B0334.2a
gag protein, putative	Bm1_16100	nDi.2.2.2.g11024	No hits.
ADP,ATP carrier protein, heart/skeletal muscle isoform T1, putative	Bm1_18515	nDi.2.2.2.g07482	T01B11.4
high mobility group protein, putative	Bm1_25620	nDi.2.2.2.g04713	Y48B6A.14
Ets-domain containing protein	Bm1_27305	nDi.2.2.2.g03657	C33A11.4a
hypothetical protein	Bm1_28665	nDi.2.2.2.g05269	ZK1236.9
LEM domain containing protein	Bm1_31790	nDi.2.2.2.g07524	W01G7.5
NifU-like protein, putative	Bm1_36610	nDi.2.2.2.g00848	Y45F10D.4
protein C33A11.1, putative	Bm1_38775	nDi.2.2.2.g01425	C33A11.1
Zinc finger, C2H2 type family protein	Bm1_38815	nDi.2.2.2.g01416	F28F9.1
LD36024p, putative	Bm1_40370	nDi.2.2.2.g03620	F53B3.5

^(a)^Reciprocal best blast matches with *B. malayi* in all cases except Y48B6A.14/Bm1_25620.

### The expression profile of predicted target mRNAs correlates inversely with that of *bpa-mir-5364*

**Figure 4:**
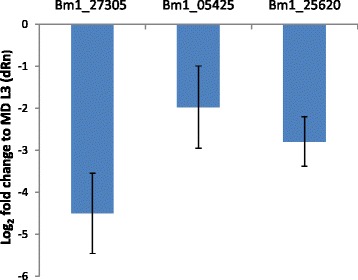
Expression profiles of three predicted mRNA targets are inversely correlated with *bpa-miR-5364*. Graph shows levels of expression of Bm1_27305 (Ets-domain containing protein), Bm1_05425 (Zinc finger N-recognin family protein) and Bm1_25620 (high mobility group protein) in day 10 post-infective L4 compared to mosquito-derived L3. As *B. pahangi* material was used throughout, protein-coding genes with identifiers of the form “Bm1_00001” refer, here, to the *B. pahangi* orthologs of the *B. malayi* genes. The mean of three biological replicates is shown +/− standard deviation. Results were normalised to β tubulin and are expressed as log_2_ fold change relative to mosquito-derived (MD) L3.

If the mRNAs identified are indeed targeted by *bpa-miR-5364* a decrease in abundance would be expected as miRNA abundance increases. To examine this, qRT-PCR was performed on *B. pahangi* mosquito-derived L3 and on day 10 p.i. L4, the time point at which maximal expression of *bpa-miR-5364* was observed, using *B. pahangi* β-tubulin as a normalising gene [45-47]. The abundance of all three selected target genes decreased significantly in day 10 p.i. L4 compared to mosquito-derived L3 (Figure 4). The inverse relationship between miRNA and mRNA abundance supports the bioinformatics prediction that these three transcripts are targeted by *bpa-miR-5364*. The expression profiles of each of these three predicted targets of *bpa-miR-5364* were also analysed from our transcriptomic data covering the L3 and later larval stages. An inverse relationship between miRNA and mRNA abundance was also observed here for the three target genes (Additional file 8). Additional analysis of the transcriptome data demonstrated that a further three mRNAs included in the final set of 13 *bpa-miR-5364* targets showed a pronounced decrease in abundance between mosquito-derived L3 and day 10 p.i. larvae (Additional file 8).

### *bpa-miR-5364* interacts directly with predicted mRNA targets

**Figure 5:**
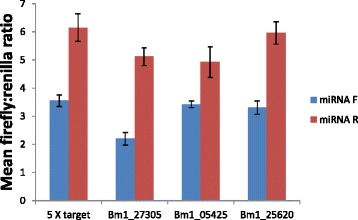
*bpa-miR-5364* interacts with predicted target gene 3′UTRs. Graph shows the mean ratio +/− standard deviation of 5–6 replicate wells comparing Firefly to Renilla luciferase signal in the presence of *miR-5364* and a synthetic gene containing five repeats of a perfect *miR-5364* binding site or the 3′UTRs of predicted target mRNAs, Bm1_05425, Bm1_27305 and Bm1_25620. Ratios using *miR-5364* cloned in the forward orientation (miRNA F) and in reverse orientation (miRNA R) are indicated. Percentage reduction in signal was calculated by comparison of the two. Figures show data from one of three representative experiments, except for plasmid Bm1_25620 which was repeated twice with comparable results.

For *Brugia* spp*.*, and related parasites, there are currently no suitable transgenic approaches or parasite-derived cell lines that could be used to directly investigate miRNA/mRNA interactions. The predicted *bpa-miR-5364*/mRNA interactions were therefore assessed using a mammalian cell system. 3′UTRs from the *B. pahangi* orthologs of Bm1_27305, Bm1_05425 and Bm1_25620 were cloned downstream of firefly luciferase and transiently transfected into HEK293 cells along with a plasmid containing *bpa-mir-5364*. Successful expression of *bpa-miR-5364* in this cell line was confirmed by qRT-PCR (results not shown). Levels of luciferase in the presence or absence of *bpa-miR-5364* were compared. For all three 3′UTRs tested, a significant reduction in expression was observed in the presence of *bpa-miR-5364*. A synthetic gene containing five copies of a perfect *bpa-miR-5364* binding site was included as a positive control in these experiments and gave a reduction in signal of 42% in the presence of *bpa-miR-5364* versus plasmid with the miRNA insert in the reverse orientation (P = 0.0142, Mann–Whitney test) in the representative experiment shown in Figure 5. For the proposed target 3′UTRs, a 57% reduction in expression was observed in the presence of *bpa-miR-5364* for Bm1_27305, a 31% reduction for Bm1_05425, and a 45% reduction for Bm1_25620 versus miRNA insert in reverse orientation (all P = 0.0051, Mann–Whitney test).

### Attempts at *bpa-miR-5364* inhibition using an antisense oligonucleotide

**Figure 6:**
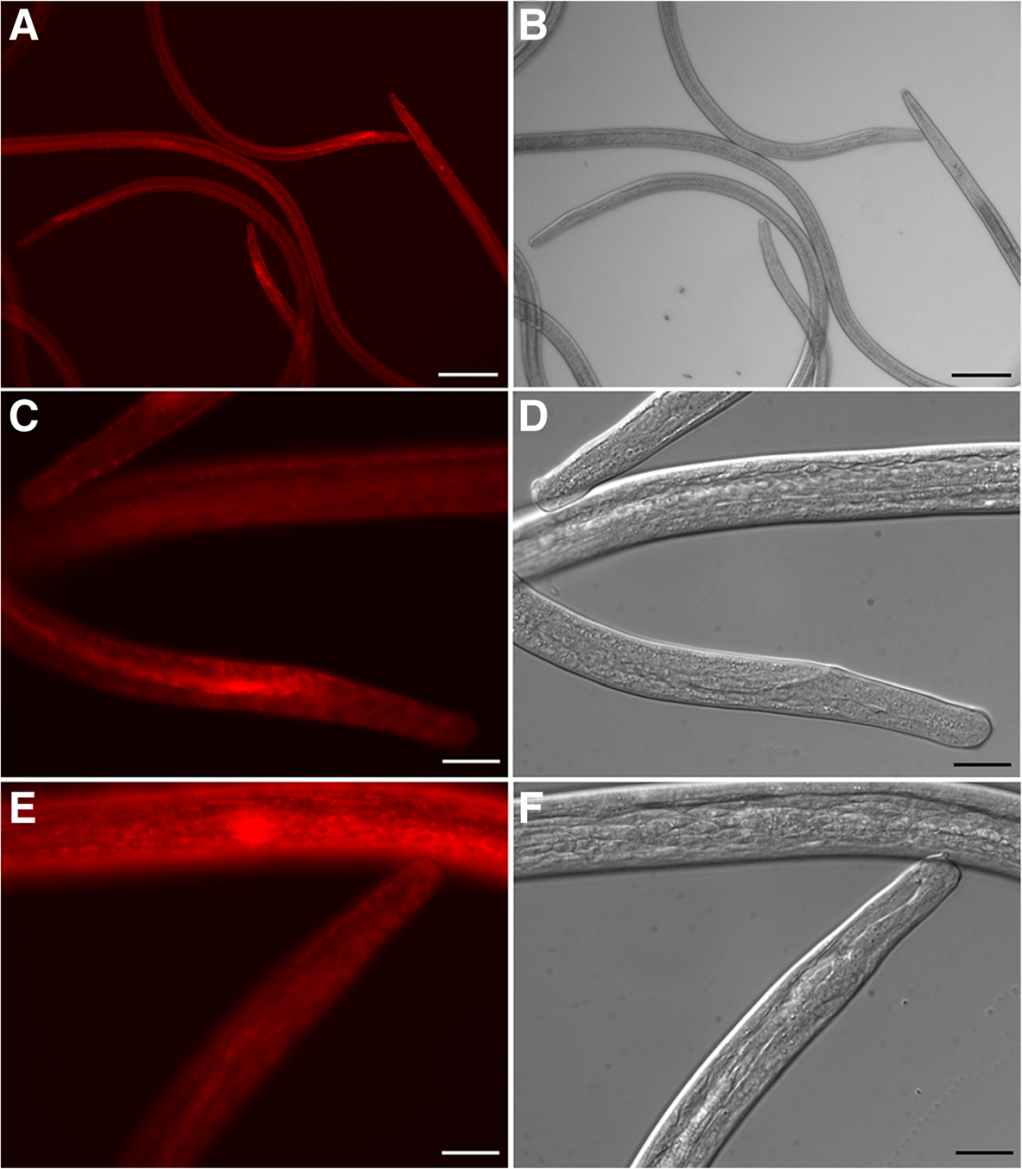
Uptake of fluorescently labelled oligonucleotides from culture medium by *B. pahangi.* Uptake of small RNAs from media was assessed after 24–48 hours of culture using 0.5 μM Cy3-labelled siRNA. **Panels A-D** show mosquito-derived L3 after 48 hours (scale bar represents 100 μm in A&B and 25 μm in C&D). **Panels E & F** show uptake in day 5 p.i. L3 after 24 hours (scale bar 25 μm). No fluorescence was observed in controls cultured in the absence of Cy3-labelled siRNA (not shown).

To examine the effect of *bpa-miR-5364* inhibition in cultured parasites, conditions for effective uptake of labelled small RNAs by *B. pahangi* larvae were first determined. Mosquito-derived L3 or day 5 p.i. L3 maintained in culture show uptake of Cy3-labeled siRNA or Cy3-labeled anti-miR (included in medium at 0.5 μM) after 24 hours and to a greater level after 48 hours culture (Figure 6). Having verified uptake, we attempted to inhibit *bpa-miR-5364* using L3 recovered from the mammalian host at 4–5 days p.i. Larvae were maintained in culture for 48 hours in the presence of an antisense RNA oligonucleotide (ASO) designed to inhibit *bpa-miR-5364* or a sequence-scrambled control oligo and levels of Bm1_27305, Bm1_05425 and Bm1_25620 mRNA assessed by qRT-PCR. However, no de-repression of any target mRNA, nor any phenotypic difference, was observed between ASO treated larvae and controls.

We also tested the effect of RNAi of the single predicted miRNA-specific argonaute gene Bm1_13960 [48] using endoribonuclease-prepared pools of complex siRNA (esiRNAs) [49-51], together with the *bpa-miR-5364* ASO. This approach is similar to that used in *C. elegans* where miRNA mutants were analysed in the background of global reduction in miRNA levels due to *alg-1* mutation [52]. This combined approach did not produce any phenotype or de-repression of target mRNAs in *B. pahangi* (not shown).

### Co-ordinated regulation of mRNA targets by multiple miRNAs

As individual mRNAs are often regulated by multiple miRNAs, this most likely explains the lack of de-repression with the *miR-5364* ASO. Therefore predictions of additional miRNAs that might function alongside *bpa-miR-5364* to target Bm1_27305, Bm1_05425 and Bm1_25620, and their *D. immitis* orthologs, were made using PITA and a set of miRNA which included all 104 mature *Brugia* spp. miRNA identified previously [14]. Predicted miRNA target sites for the three target genes in both parasite species are shown in Additional file 9. Interestingly, conserved sites for *let-7* and *miR-84* are found for Bm1_27305 with almost identical positioning in each species. Given the sequence similarity between *miR-5364* and the *let-7* family (Figure 2A) this might be expected; however, the binding sites for *miR-5364* and *let-7/miR-84* are not identically positioned (see Additional file 9). In addition, the *C. elegans* ortholog of Bm1_27305, C33A11.4 (Table 2) also known as *tag-97*, has predicted binding sites for *let-7* family miRNAs using PITA (results not shown) and TargetScan [53] (http://www.targetscan.org/worm_52/), indicating that regulation of Bm1_27305 and its orthologs by this important miRNA family may be a feature conserved throughout the nematode phylum. Similarly, using PITA the *C. elegans* ortholog of Bm1_05425 (T22C1.1) has a site for the *let-7* family member *miR-794* (results not shown). Predicted binding sites for the orthologous miRNAs *bantam* and *mir-82* [14] are found for Bm1_25620 (Additional file 9) and in the TargetScan predictions for the *C. elegans* ortholog (Y48B6A.14, hmg-1.1), suggesting conservation of miRNA-mediated regulation across species. Further work will reveal if these interactions are functional and if these miRNAs act in conjunction with *miR-5364* to regulate its targets.

## Discussion

Infection of the mammalian host by the L3 stage of filarial worms is a key event in the life cycle: how the parasite survives and differentiates within its new environment is poorly understood. Here we show that *bpa-miR-5364*, which is specific to clade III parasitic nematodes and related to the *let-7* family, is highly up-regulated to coincide with transmission to the mammalian host and may be involved in the infection event by targeting specific mRNAs. The L3 of many parasitic nematodes show parallels with the developmentally arrested dauer larva of *C. elegans*. In a recent study, four *C. elegans* miRNAs were found to be up-regulated in samples of mixed stages compared to dauers, two of which are the *let-7* family members *miR-241* and *miR-795* [26]. Similarly, in a detailed analysis of miRNA expression following dauer or continuous development, *let-7* family members showed increased expression post-dauer [54]. *Brugia* spp. possess three *let-7* family members, *let-7*, *miR-84* and *miR-5364,* all of which are up-regulated post-infection, with expression of *miR-5364* increasing first, within 24 hours, and rising to a significantly greater level than either *let-7* or *miR-84*, suggesting that in *Brugia* spp. *miR-5364* has a dominant role. We therefore aimed to identify the possible functions of this miRNA by identifying its target genes.

Identifying mRNA targets is complex because most bioinformatics tools search primarily for complementarity between the short miRNA seed sequence (5′ nucleotides 2–7) and the mRNA, and then take other factors into account, including the free energy of the miRNA-mRNA duplex and position of binding within the 3′UTR. Thus, bioinformatics tools potentially identify hundreds of possible targets, many of which may not be verifiable. In an attempt to overcome this problem, the availability of other filarial genomes was exploited in a comparative genomics approach. We identified orthologous mRNAs in different clade III nematodes that have conserved binding sites for *miR-5364*, with the further requirement that the site be similarly positioned. This approach, which has been used previously in vertebrates and nematodes [53,55], significantly reduced the number of possible target mRNAs from over 850 to 13. Three of these were selected for in-depth study: Bm1_27305 encoding an ETS domain transcription factor, Bm1_05425 encoding an N-recognin-type zinc finger protein, and Bm1_25620 which encodes a putative high mobility group protein. All three genes were found to have a reciprocal expression profile to *bpa-miR-5364,* both from analysis of transcriptomic data and by qRT-PCR, consistent with miRNA-mediated negative regulation. To demonstrate a direct interaction between miRNA and target we used transient transfection of mammalian HEK293 cells and a dual luciferase assay system and found that the presence of *bpa-miR-5364* resulted in the down-regulation of the luciferase reporter gene for all three 3′UTRs, providing further evidence that these genes are true targets of this miRNA.

All three verified targets of *bpa-miR-5364* could potentially function to alter gene and/or protein levels and a decrease in the levels of these post-infection may be important for larval activation and/or developmental progression in the mammalian host. The human ortholog of Bm1_27305 (EHF, NCBI Gene ID: 26298) belongs to the ETS domain transcription factor family, which are characterised by epithelial-specific expression (reviewed in [56]) and by tight tissue-specific and temporal regulation. For example, in prostate cancer this transcription factor controls the balance between cell proliferation and differentiation [57]. The *C. elegans* homolog of Bm1_27305 (C33A11.4) is upregulated in mutants of the miRNA-specific argonaute gene *alg-1* [58] and is enriched in AIN-1 (ALG-1 INteracting protein) pull-downs [59], consistent with miRNA regulation. Bm1_05425 contains an N-recognin zinc finger motif conserved in ubiquitin-ligases, which target proteins for degradation. In *C. elegans*, an important function of *let-7* is the negative regulation of LIN-41 [3,11], which contains a ubiquitin ligase domain. In turn, LIN-41 negatively regulates levels of the transcription factor LIN-29, which is required for adult cell fates [3,11]. It is possible that in *Brugia* spp. L3*,* Bm1_05425 may perform a similar function to LIN-41 in *C. elegans*, as proteins with ubiquitin ligase activity could potentially target a subset of proteins for degradation. The final *mir-5364* target studied, Bm1_25620, is predicted to encode an HMG protein. These bind to chromatin and can act in the regulation of transcription during development, DNA repair, apoptosis or cell senescence [60]. In mammalian cells, HMGA2 is abundant during early development and levels decrease during later stages of development and differentiation, via *let-7* regulation [61]. A reduction in HMG Bm1_25620 following transmission to the mammalian host may allow larval development and/or exit from an arrested state.

It is important to note that whatever the function of *bpa-miR-5364*, it is not likely to be restricted to the L3 stage, as in the life cycle stages tested, expression peaked in day 10 p.i. L4, an actively growing stage and continued into adulthood. Whether the target mRNAs are the same between the p.i. L3 and subsequent developmental stages of *Brugia* spp. is currently unknown. Even in *C. elegans* where miRNAs are comparatively well defined, little is known of target conservation between different life cycle stages.

In an attempt to define the possible function of the mRNA targets in *B. pahangi*, we tested the effects of inhibiting *bpa-miR-5364* in the larval stages using a specific ASO. ASOs bind with sequence complementarity to the mature miRNA to disrupt binding to mRNA targets. While we found efficient uptake of labelled RNA by *B. pahangi* L3 and p.i. L3s in the culture conditions tested, no de-repression of any of the three verified target genes was found using the ASO. As loss of a single miRNA is often not sufficient to reveal function, we also tested the effect of the *mir-5364* ASO along with siRNAs targeting the *Brugia* spp. miRNA-specific argonaute, but found no observable effect. Therefore, it may be necessary to include specific inhibitors of additional miRNAs to reveal the effect of *bpa-miR-5364* disruption. However, the conditions required for inhibition of miRNA function in parasitic nematodes are completely unknown, with this being the first study to attempt to inhibit miRNAs. Factors such as the concentration of ASO required, efficiency of uptake in different cells and tissues, and the ability of exogenously applied inhibitors to enter the RISC and block target binding will all influence the ability to inhibit miRNA function.

As expression of *bpa-miR-5364* increased 12-fold between vector derived L3 and 24 h p.i.L3, it was of interest to determine what factors might induce the expression of this miRNA. Previous studies on the control of gene expression in vector-derived L3 versus 24 hour p.i. L3 highlighted a number of mRNAs which appeared to be regulated largely by the temperature shift associated with infection [62]. By simply moving L3 to 37°C in an atmosphere of 5% CO_2_ in air, the expression of *bpa-miR-5364* was induced, with serum having minimal additional effect. Recent studies in *Arabidopsis* have shown that increased temperature and CO_2_ lead to significant changes in expression of several miRNAs [63], but whether temperature or CO_2_ has any role in the induction of *bpa-mir-5364* requires further study. In addition, autoregulation of *let-7* has been identified in *C. elegans*, in which binding of *let-7* to its own 3′UTR acts to increase processing of the pri-miRNA by argonaute, leading to higher levels of the mature miRNA [21]. Similarly, binding of *C. elegans lin-4* to a site in its own promoter activates *lin-4* transcription [22]. In this study, we identified potential *bpa-miR-5364* binding sites in genomic sequence upstream and downstream of the mature miRNA region, suggesting that autoregulation could also be important for control of this parasite miRNA.

*mir-5364* appears to be restricted to clade III nematodes as, to date, related sequences have not been found in other parasitic or free-living nematode genomes. In addition to *mir-5364* in *Brugia* spp. [14,15], we found this miRNA in the genomes of five additional clade III filarial parasites, with a recent study confirming expression in *D. immitis* [64]. *mir-5364* is not restricted to filarial nematodes as it is also expressed in the clade III gastrointestinal nematode *A. suum* [24] suggesting its role is not specific to tissue-dwelling nematodes. Therefore *mir-5364* may have diverged from the *let-7* family during the evolution of clade III nematodes, an event that occurred 350–540 million years ago [65]. These observations support the concept that small RNA sequence and function have evolved and diversified between nematode clades, as suggested previously following the identification of 21U (or piwi-interacting) RNAs in clade V nematodes, but not, to date, in clade III [14,24] or clade IV [29] nematodes.

## Conclusions

In conclusion, we report a *B. pahangi* miRNA that is highly up-regulated to coincide with the transition between hosts and identify some of its mRNA targets. *bpa-miR-5364* is a novel member of the *let-7* family, consistent with the function of other members of that family in nematode development. Infection of the mammalian host is the key event in the filarial life cycle and our data indicate that post-transcriptional regulation of gene expression may be necessary for this event.

## Methods

### *B. pahangi* maintenance and culture

*B. pahangi* was maintained as described previously [14,66]. For developmental gene expression analysis, mosquito-derived L3 were frozen immediately following isolation from the mosquito, while p.i. larval stages and adult worms were collected by peritoneal lavage of infected jirds at specific time-points. *B. pahangi* larvae for culture were prepared as described previously [62] and cultured in Worm Culture Medium (WCM) (RMPI 1640 with glutamine and HEPES, supplemented with 100 units/ml of penicillin and 100 μg/ml streptomycin, 5% heat inactivated fetal calf serum (FCS), and 1% glucose, all from Invitrogen) at 37°C in 5% CO_2_/air. All animal protocols were carried out in accordance with the guidelines of the UK Home Office, under the Animal (Scientific Procedures) Act 1986, following approval by the University of Glasgow Ethical Review Panel. Experiments were performed under the authority of the UK Home Office, project numbers 60/4448 and 60/3792.

### Total RNA extraction and miRNA microarray analysis

Total RNA was extracted using the Trizol protocol (Invitrogen) after manual disruption, with RNA for miRNA microarray prepared with an additional 75% ethanol wash. Biological replicates were defined as parasites isolated from different animals, or, for mosquito-derived L3, extracted at different times. Microarrays were performed by a service provider (LC Sciences) using a custom array containing probes to 252 *Brugia* spp. sequences (all the mature and star miRNA sequences identified in our previous study [14] with three additional sequences found by others [19]), and all *C. elegans* sequences present in miRBase release 15 (ftp://mirbase.org/pub/mirbase/15/).

### miRNA and mRNA qRT-PCR

For miRNA qRT-PCR, the miRNA 1^st^ Strand cDNA Synthesis protocol (Agilent Technologies) was followed using 1 μg of DNase I-treated (Ambion) total RNA for the polyadenylation reaction and enzyme-plus and enzyme-minus reverse transcription reactions. qPCR was performed following the miRNA qPCR Master Mix protocol (Agilent Technologies). Results were normalised to *bpa-miR-100c*, which was found by microarray to be present at similar levels throughout the developmental stages examined. For mRNA qRT-PCR, 1 μg of DNase I-treated total RNA was used in enzyme-plus and enzyme-minus reverse transcriptions using the SuperScript II (Invitrogen) protocol with oligo(dT) primer. mRNA qRT-PCR was performed following the Brilliant III Ultra-Fast SYBR Green QPCR Master Mix (Agilent Technologies) protocol using β-tubulin (Genbank AY705382) as a normalising gene. For both miRNA and mRNA qRT-PCR, as well as biological triplicate samples, each PCR was carried out in triplicate and run on an Agilent Mx3005P qPCR System. The sequences of the HPLC-purified oligonucleotides used are given in Additional file 10.

### Induction of *bpa-miR-5364* in cultured parasites

Four sets of 500 L3 were isolated from mosquitoes and prepared for culture as described above. One set was frozen immediately while the other three sets were cultured at 37°C, 5% CO_2_ in air in WCM adjusted to contain either no FCS, 5% FCS, or 10% FCS. After 24 hours the larvae were collected and frozen. Total RNA was extracted as described above and the entire preparation DNAase I-treated (Ambion), ethanol precipitated and used for qRT-PCR.

### Bioinformatic prediction of *miR-5364* targets

The *B. malayi* genome [35] was downloaded from the following URL, http://www.ncbi.nlm.nih.gov/nuccore?term=DS236884:DS264093[PACC] as genomic scaffolds. From the same site the annotated genome was downloaded in Genbank(full) format then converted to GFF using ReadSeq (http://iubio.bio.indiana.edu/soft/molbio/readseq/java/). *B. malayi* proteins sequences were downloaded from http://ghedinlab.csb.pitt.edu/GhedinLab/Parasite%20Genomics?action=AttachFile&do=view&target=brugia_aa_112707.fa.gz. The *Dirofilaria immitis* genome [36] version 2 reassembly files “Dirofilaria_immitis_2.2”, “nDi.2.2.2.aug.blast2go”, and “nDi.2.2.2.aug.proteins” were downloaded from http://badger.bio.ed.ac.uk/filarial/home/download. Use of this updated version of the published *D. immitis* genome was kindly provided pre-publication by Sujai Kumar and colleagues. Sequences 1 kb 3′ of the annotated stop codon for all genes were extracted for both species. The miRNA target prediction algorithms PITA [23] (http://genie.weizmann.ac.il/pubs/mir07/mir07_exe.html) and miRanda [41], (http://www.microrna.org/microrna/getDownloads.do) were run as standalone programs (using default settings) on the 3′ downstream sequence sets from each species searching for *mir-5364* sites. PITA default settings permit seeds of the following types, 6:0:0, 7:0:1, 8:1:1 (seed length: number of G-U pairings allowed in seed: number of mismatches allowed in seed). For each species the PITA and miRanda predictions were combined and only target predictions found by both methods were retained. The predicted protein sets for *B. malayi* and *D. immitis* were analysed using InParanoid [42] (http://inparanoid.sbc.su.se/cgi-bin/faq.cgi) to identify orthologs. Reciprocal BLASTP analysis at NCBI (http://www.blast.ncbi.nlm.nih.gov/Blast.cgi?PROGRAM=blastp&PAGE_TYPE=BlastSearch&LINK_LOC=blasthome) was used to identify the orthologs of *B. malayi* genes in *L. loa* and *W. bancrofti*, while for *L. sigmodontis* one-way TBLASTN was performed at 959 Nematode Genomes (http://xyala.cap.ed.ac.uk/downloads/959nematodegenomes/blast/blast.php). To test for the presence of *mir-5364* binding sites, sequence 1 kb downstream from the annotated stop of each gene was analysed using PITA online (http://genie.weizmann.ac.il/pubs/mir07/mir07_prediction.html, with settings: 7 nucleotide seed, no mismatch allowed, 1 G:U allowed). Reciprocal BLAST analysis was used to identify the *C. elegans* orthologs.

### Prediction of additional miRNAs targeting Bm1_27305, Bm1_05425 and Bm1_25620

The downstream sequences of Bm1_27305, Bm1_05425 and Bm1_25620 and their *D. immitis* orthologs were analysed for the presence of miRNA binding sites using the set of 252 *Brugia* spp. miRNA sequences (described above) with PITA online. Settings for *Brugia* spp. were as follows: 7 nucleotide seed, 0 mismatch allowed, 1 G-U allowed; and for *D. immitis* 7:1:1 to compensate for potential miRNA sequence variation. Only those sites with PITA ΔΔG energies of < −7 and located within the longest experimentally defined 3′UTR (see below) were considered.

### 3′RACE analysis of predicted mRNA targets of *bpa-miR-5364*

mRNA 3′ ends were analysed using the 3′ RACE System for Rapid Amplification of cDNA Ends (Invitrogen) following the manufacturer’s protocol. 1 μg of DNase I (Ambion) treated mosquito-derived *B. pahangi* L3 total RNA was used along with the gene-specific primers designed to *B. malayi* sequences (primers in Additional file 10). 3’ RACE products were cloned into pCR2.1-TOPO (Invitrogen) and sequenced. The longest experimentally defined 3′UTRs were 418, 182 and 504 nucleotides downstream of the annotated stop for Bm1_27305, Bm1_05425, and Bm1_25620 respectively.

### RNAseq library construction and sequencing

*B. pahangi* RNA isolated from mosquito-derived L3, day 1, day 5 and day 10 p.i. larvae was used to generate standard Illumina RNAseq libraries with two biological replicates. Libraries were sequenced using the Illumina HiSeq 2000 platform as 100 bp pair-end sequences. Sequencing reads were submitted to the European Nucleotide Archive (experiment accession number: ERX199547; http://www.ebi.ac.uk/ena/data/view/ERX199547).

### Mapping of sequencing reads and differential expression analysis

Mapping of sequencing reads was performed using Tophat version v2.0.8b [67] with default parameters except for “-g 1 --library-type fr-unstranded -r 200 --mate-std-dev 100 -a 6 -i 10 -I 40000 --microexon-search --min-segment-intron 10 --max-segment-intron 40000”. The *B. pahangi* draft genome version 1.5.4, available through Wormbase ParaSite, was used as reference (http://parasite.wormbase.org/Brugia_pahangi_prjeb497/). Alignment files resulting from the mapping step were further sorted and indexed using SAMtools version: 0.1.19-44428 cd [68] using the sort and index functions respectively. Counts per transcript were retrieved using the htseq-count module from the HTSeq package version 0.6.0 [69] with default parameters except for “-f bam -a 30 -t exon -s no -m union”. The reference annotation used in this analysis is the version WBPS1 available through Wormbase ParaSite. Differential expression analysis was performed using the R language package EdgeR version 3.4.2 [70]. Normalised counts plots were generated using ggplot2 version 1.0.0.

### Analysis of miRNA/mRNA interactions in HEK293 cells

3′UTRs from Bm1_27305, Bm1_05425 and Bm1_25620 were amplified from *B. pahangi* genomic DNA (isolated as described previously [50]) using PfuUltra II Fusion HS DNA Polymerase (Agilent) (primers in Additional file 10). Products were cloned into pCR2.1-TOPO (Invitrogen) then subcloned into the *Not* I site downstream of firefly luciferase in pMir-Target (Origene) to generate plasmids pABR212 (Bm1_27305 3′UTR in pMir-Target), pABR210 (Bm1_05425 3′UTR in pMir-Target) and pABR211 (Bm1_25620 3′UTR in pMir-Target). A construct containing five repeats of the perfect target sequence for *bpa-miR-5364* was synthesised by GeneArt (Invitrogen) (sequence in Additional file 10) and cloned into pMir-Target using *Eco* RI and *Not* I to generate plasmid pMir-T:Bp-mir-5364-5Xtarget. A 317 bp section of the *bpa-mir-5364* locus was amplified from *B. pahangi* genomic DNA (primers in Additional file 10), cloned into pCR2.1-TOPO and subcloned into vector pEGFP-N1 (Clontech) using *Kpn* I to generate plasmids pABR213 (*bpa-mir-5364* in vector pEGFP-N1, sense orientation) and pEGFPN1:Bp-mir-5364-Rev (*bpa-mir-5364* in vector pEGFP-N1, reverse orientation).

HEK293 cells were maintained in Dulbecco's Modified Eagle's Medium (containing 4500 mg/L glucose and sodium bicarbonate, Sigma D5671) (supplemented with 2 mM L-Glutamine, 100 units/ml of penicillin, 100 μg/ml streptomycin, and 10% heat inactivated FCS, all from Invitrogen) at 37°C in 5% CO_2_/air. For transfections, 2 × 10^4^ cells/well were seeded into the wells of 96-well plates in a volume of 100 μl using the above growth medium but without addition of antibiotics. Cells were transfected after 24 hours when they were ~60% confluent using Lipofectamine LTX (Invitrogen) with 50 ng of a *mir-5364-*containing plasmid, 6.25 ng of a pMir-Target-derived plasmid and 0.5 ng of phRG-TK (Renilla luciferase, Promega). Transfections were performed following the manufacturer’s protocol for HEK293 cells. DNA was diluted to a volume of 20 μL using Opti-MEM I Reduced Serum Medium (Invitrogen) and 0.35 μL Lipofectamine LTX Reagent added. After incubation at room temperature for 30 minutes 20 μL of the DNA-Lipofectamine complex was then added directly to the cells in each well. Cells were grown for 48 hours then analysed using a Dual Luciferase Assay kit (Promega) following the manufactures protocol with six replicates used per test condition.

### miRNA inhibition

Conditions for uptake of double-stranded and single-stranded RNA from medium by *B. pahangi* were tested using Silencer Cy3 Labeled Negative Control No. 1 siRNA (Ambion) and Cy3 dye-Labeled Anti-miR Negative Control No. 1 (Ambion) at a final concentration of 0.5 μM. ~100 mosquito-derived L3 and ~30 day 5 p.i. L3, both prepared as described above, were added to 0.5 ml WCM (5% FCS) in a 48-well culture plate, with and without the addition of Cy3-siRNA and Cy3-anti-miR, and uptake analysed after 24 and 48 hours. An antisense oligonucleotide (ASO) [71] to inhibit *bpa-mir-5364* was purchased from Eurofins MWG Operon along with a scrambled version of the ASO (sequences in Additional file 10). The ASOs consisted of all 2’O-methyl RNA bases, phosphorothioate bonds at the 5′ and 3′ ends, as well as nucleotides added at each end. The *B. pahangi* ortholog of the full-length predicted Bm1_13960 (argonaute) sequence was amplified from mosquito-derived *B. pahangi* cDNA using PfuUltra II Fusion HS DNA Polymerase (Agilent) (primers in Additional file 10). A ~1.7 kb *Pst* I/*Pst* I fragment of this was cloned into pPD129.36 (Addgene) to create plasmid pADW0062 which was linearised with *Not* I and *Hind* III. *In vitro* transcription and preparation of endoribonuclease-prepared pools of complex siRNA (esiRNA) was carried out as described previously [50]. Larvae were harvested at either 4 or 5 days p.i. and cultured in 24-well plates containing 1 ml WCM with ~150-200 larvae per well. Larvae were treated under three conditions, 1) 5 μM *mir-5364* ASO plus 1 μM Bm1_13960 (argonaute) esiRNA, 2) 5 μM scrambled ASO plus 1 μM Bm1_13960 esiRNA, and 3) medium only. Each condition was replicated in three wells and the experiment repeated twice. Larvae were cultured for 48 hours, frozen at −80°C and analysed by qRT-PCR.

### Microscopy

*B. pahangi* larvae were prepared for microscopy by washing three times in 1 ml of PBS/0.1% Tween in 1.5 ml tubes, using centrifugation at 2000 rpm in a benchtop microfuge for 2 minutes. After the final wash, the volume was reduced to ~20 μL and 10 μL pipetted onto microscope slides with a 2% agarose pad. Images were collected using an Axioskop 2 Plus microscope (Zeiss), ORCA-ER digital camera (Hamamatsu) and Openlab (Improvision) software.

## Additional files

Additional file 1:
**miRNA microarray data.**
Additional file 2:
**Pairwise comparison of larval stage microarray results.**
Additional file 3:
***mir-5364*** **in five clade III parasitic nematode genomes.**
Additional file 4:
**miPred analysis of** ***mir-5364*** **from five clade III parasitic nematodes.**
Additional file 5:
***Brugia*** **and** ***D. immitis miR-5364*** **target predictions.**
Additional file 6:
**Orthologous** ***miR-5364*** **targets in** ***Brugia*** **and** ***D. immitis.***
Additional file 7:
**Predicted** ***miR-5364*** **target sites in orthologs of Bm1_ 27305 & Bm1_05425.**
Additional file 8:
**Expression profiles of predicted** ***bpa-miR-5364*** **targets by RNAseq.**
Additional file 9:
**Additional miRNAs targeting Bm1_27305, Bm1_05425 &Bm1_25620.**
Additional file 10:
**Oligonucleotide primer and synthetic gene sequences.**
