# Supplementary material for: A novel member of the let-7 microRNA family is associated with developmental transitions in filarial nematode parasites
Source: BMC Genomics. 2015 Apr 22;16(1):331. doi: 10.1186/s12864-015-1536-y (PMC4428239; doi:10.1186/s12864-015-1536-y)
Supplement: Additional file 2: — Pairwise comparison of larval stage microarray results. [file 12864_2015_1536_MOESM2_ESM.docx]

**Microarray pairwise comparison of larval stages.**

**A) Mosquito-derived L3 versus day 1 p.i. L3**

|  |  | **MD L3 ^(a)^** | **Day 1 p.i L3** |  |
| --- | --- | --- | --- | --- |
| **Reporter Name** | **p-value** | **Mean** | **Mean** | **Log_2_ (day 1/ MD L3)** |
| *bpa-miR-5364* | 6.29E-04 | 708 | 8,794 | 3.64 |
| *cel-miR-51* | 4.92E-03 | 3,055 | 173 | -4.14 |
| *cel-miR-266* | 9.96E-03 | 607 | 20 | -4.90 |

**B) Mosquito-derived L3 versus day 5 p.i. L3**

|  |  | **MD L3** | **Day 5 p.i L3** |  |
| --- | --- | --- | --- | --- |
| **Reporter Name** | **p-value** | **Mean** | **Mean** | **Log_2_ (day 5/ MD L3)** |
| *bpa-let-7* | 2.78E-04 | 93 | 2,713 | 4.87 |
| *bpa-miR-84-5p* | 1.41E-03 | 4 | 409 | 6.67 |
| *bpa-miR-5364* | 2.13E-03 | 708 | 14,922 | 4.40 |
| *cel-let-7* | 4.49E-03 | 80 | 2,831 | 5.15 |
| *bpa-miR-5853** | 7.05E-03 | 310 | 40 | -2.95 |
| *cel-miR-74* | 8.44E-03 | 31 | 7 | -2.19 |

**C) Mosquito-derived L3 versus day 10 p.i. L4**

|  |  | **MD L3** | **Day 10 p.i L4** |  |
| --- | --- | --- | --- | --- |
| **Reporter Name** | **p-value** | **Mean** | **Mean** | **Log_2_ (day 10/ MD L3)** |
| *bpa-miR-5364* | 2.59E-04 | 708 | 17,301 | 4.61 |
| *bpa-let-7* | 5.16E-04 | 93 | 3,068 | 5.05 |
| *bpa-miR-84-5p* | 8.94E-04 | 4 | 669 | 7.38 |
| *cel-miR-266* | 3.04E-03 | 607 | 24 | -4.64 |
| *Bpa0107* | 4.66E-03 | 3,852 | 417 | -3.21 |
| *cel-let-7* | 5.32E-03 | 80 | 3,130 | 5.29 |
| *cel-miR-789* | 7.06E-03 | 808 | 48 | -4.07 |
| *bpa-miR-5853* | 7.91E-03 | 1,329 | 275 | -2.27 |

**D) Day 1 p.i. L3 versus day 5 p.i. L3**

|  |  | **MD L3** | **Day 5 p.i** |  |
| --- | --- | --- | --- | --- |
| **Reporter Name** | **p-value** | **Mean** | **Mean** | **Log_2_ (day 5/day 1)** |
| *bpa-let-7* | 9.25E-04 | 214 | 2,713 | 3.67 |
| *cel-let-7* | 2.86E-03 | 170 | 2,831 | 4.05 |
| *bpa-miR-84-5p* | 3.48E-03 | 18 | 409 | 4.51 |
| *bpa-miR-84-3** | 3.85E-03 | 20 | 402 | 4.36 |

**E) Day 1 p.i. L3 versus day 10 p.i. L4**

|  |  | **MD L3** | **Day 10 p.i** |  |
| --- | --- | --- | --- | --- |
| **Reporter Name** | **p-value** | **Mean** | **Mean** | **Log_2_ (day 10/day 1)** |
| *bpa-let-7* | 1.46E-03 | 214 | 3,068 | 3.84 |
| *bpa-miR-84-5p* | 1.87E-03 | 18 | 669 | 5.22 |
| *bpa-miR-84-3** | 2.69E-03 | 20 | 634 | 5.02 |
| *cel-let-7* | 3.18E-03 | 170 | 3,130 | 4.20 |
| *bpa-miR-5872* | 6.29E-03 | 32 | 194 | 2.60 |

^(a)^ Mosquito-derived L3.
