# Supplementary material for: A novel member of the let-7 microRNA family is associated with developmental transitions in filarial nematode parasites
Source: BMC Genomics. 2015 Apr 22;16(1):331. doi: 10.1186/s12864-015-1536-y (PMC4428239; doi:10.1186/s12864-015-1536-y)
Supplement: Additional file 3: — mir-5364 in five clade III parasitic nematode genomes. [file 12864_2015_1536_MOESM3_ESM.docx]

***mir-5364* in five clade III parasitic nematode genomes.**

| **Species and contig** | **Co-ords** | **Sequence (mature miRNA in uppercase)** | **Structure, produced using mfold** (<http://mfold.rit.albany.edu/?q=mfold/RNA-Folding-Form>) |
| --- | --- | --- | --- |
| *Dirofilaria immitis* [DimmContig6509](http://xyala.cap.ed.ac.uk/downloads/959nematodegenomes/blast/getcontig.pl?DB=Dirofilaria_immitis_v1.3_20110901.fna&CONTIG=DimmContig6509) | 309-228 | >Dim-mir-5364_predicted  ggttaagcgattcagctaataaacgttacttcagcttgattgcatataaatgcCGAGGTATTGTTTATTGGCTGAatgctat | 10 20 30 40  gguua g u a u------\| a  agc auucagcuaauaaacg uacuuc gc ug u  ucg uaAGUCGGUUAUUUGU AUGGAG cg ac u  ua--- - U C uaaauau^ g  80 70 60 50 |
| [gi\|285857107\|gb\|ADBU01001969.1\|](http://xyala.cap.ed.ac.uk/downloads/959nematodegenomes/blast/getcontig.pl?DB=Loa_loa_GenBank_27102010.fna&CONTIG=gi%7C285857107%7Cgb%7CADBU01001969.1%7C) *Loa loa* cont1.1969 | 1492-1580 | >Llo-mir-5364_predicted  ggttaagcagttcagctaataaacgctacttctgcttgatgcatataggtgcCGAGGTATTGTTTATTGGCTGAgtgctatacctccag | 10 20 30 40  ----- ua g c --\| aug  ggu agca uucagcuaauaaacg uacuuc ugcuug \  cca ucgu gAGUCGGUUAUUUGU AUGGAG guggau c  gaccu ua - U Cc^ aua  80 70 60 50 |
| [gi\|285834334\|gb\|ADBV01010407.1\|](http://xyala.cap.ed.ac.uk/downloads/959nematodegenomes/blast/getcontig.pl?DB=Wuchereria_bancrofti_GenBank_27102010.fna&CONTIG=gi%7C285834334%7Cgb%7CADBV01010407.1%7C) *Wuchereria bancrofti* cont1.10407 | 72-208  1626-1762  (two identical sequences on the same contig) | >Wba-mir-5364_predicted  ttcttctttccattctgttcctttcaccgtcagttgtagtggtggttaagcaattcagctaataaacactacttctgcttgatgcgtacaggtgcCGAGGTATTGTTTATTGGCTGAgtgctatatctccagaattc | 40 50 60 70 80  uag--- u ua a c -- aug  ugg ggu agca uucagcuaauaaaca uacuuc ugcuug \  acc cua ucgu gAGUCGGUUAUUUGU AUGGAG guggac c  cuuaag u ua - U Cc aug  130 120 110 100 90 |
| *Litomosoides sigmodontis* [331415](http://xyala.cap.ed.ac.uk/downloads/959nematodegenomes/blast/getcontig.pl?DB=Litomosoides_sigmodontis_abyss_1.fna&CONTIG=331415) | 1438-1522 | >Lsi-mir-5364_predicted  ggttaagcaattagctaatgaacgctacttctgcttgattcatatgaatgcCGAGGTATTGTTTATTGGCTGAatgctacacctc | 10 20 30 40  --\| ua a c u uug a  ggu agca uuagcuaaugaacg uacuuc gc auuc u  cca ucgu AGUCGGUUAUUUGU AUGGAG cg uaag a  cu^ ca a U C --- u  80 70 60 50 |
| *Onchocerca ochengi* [contig_10156](http://xyala.cap.ed.ac.uk/downloads/959nematodegenomes/blast/getcontig.pl?DB=Onchocerca_ochengi_v0.3_20110126.fna&CONTIG=contig_10156) | 7407-7484 | >Ooc-mir-5364_predicted  ttaagcgattcagctaatgaacgttacttcagcttgatatatagaaatgcCGAGGTATTGTTTATTGGCTGAatgcta | 10 20 30 40  uua\| g u a uugauau  agc auucagcuaaugaacg uacuuc gc \  ucg uaAGUCGGUUAUUUGU AUGGAG cg a  a--^ - U C uaaagau  70 60 50 |
