# Supplementary material for: A novel member of the let-7 microRNA family is associated with developmental transitions in filarial nematode parasites
Source: BMC Genomics. 2015 Apr 22;16(1):331. doi: 10.1186/s12864-015-1536-y (PMC4428239; doi:10.1186/s12864-015-1536-y)
Supplement: Additional file 4: — miPred analysis of mir-5364 from five clade III parasitic nematodes. [file 12864_2015_1536_MOESM4_ESM.docx]

**miPred analysis of *mir-5364* from five clade III parasitic nematodes.**

miPred (<http://www.bioinf.seu.edu.cn/miRNA/index.html>).

| Sequence Name: | *Dim-mir-5364*_predicted | *Llo-mir-5364*_predicted | *Wba-mir-5364*_predicted (nucleotides 37-137) | *Lsi-mir-5364*_predicted | *Ooc-mir-5364*_predicted |
| --- | --- | --- | --- | --- | --- |
| Species: | *D. immitis* | *Loa loa* | *W. bancrofti* | *L. sigmodontis* | *O. ochengi* |
| Sequence Content: | GGUUAAGCGAUUCAGCUAAUAAACGUUACUUCAGCUUGAUUGCAUAUAAAUGCCGAGGUAUUGUUUAUUGGCUGAAUGCUAU | GGUUAAGCAGUUCAGCUAAUAAACGCUACUUCUGCUUGAUGCAUAUAGGUGCCGAGGUAUUGUUUAUUGGCUGAGUGCUAUACCUCCAG | UAGUGGUGGUUAAGCAAUUCAGCUAAUAAACACUACUUCUGCUUGAUGCGUACAGGUGCCGAGGUAUUGUUUAUUGGCUGAGUGCUAUAUCUCCAGAAUUC | GGUUAAGCAAUUAGCUAAUGAACGCUACUUCUGCUUGAUUCAUAUGAAUGCCGAGGUAUUGUUUAUUGGCUGAAUGCUACACCUC | UUAAGCGAUUCAGCUAAUGAACGUUACUUCAGCUUGAUAUAUAGAAAUGCCGAGGUAUUGUUUAUUGGCUGAAUGCUA |
| Length: | 82 | 89 | 101 | 85 | 78 |
| Pre-miRNA-like Hairpin? | Yes | Yes | Yes | Yes | Yes |
| The Secondary Structure: | .....(((.((((((((((((((((.((((((.((................)).)))))).))))))))))))))))))).. | (((..((((.(((((((((((((((.((((((.((...............)).)))))).)))))))))))))))))))..)))..... | ...(((.(((..((((.(((((((((((((((.((((((.(((((.......)))))...)))))).)))))))))))))))))))..))).)))...... | (((..((((.((((((((((((((.((((((.((...((((....)))))).)))))).)))))))))))))).))))..))).. | ...(((.((((((((((((((((.((((((.((...............)).)))))).))))))))))))))))))). |
| MFE: | -32.99 | -36.36 | -40.50 | -35.70 | -33.16 |
| p-value (shuffle times:1000) | 0.001 | 0.001 | 0.001 | 0.001 | 0.001 |
| Prediction result: | Real microRNA precursor | Real microRNA precursor | Real microRNA precursor | Real microRNA precursor | Real microRNA precursor |
| Prediction confidence: | 74.4% | 84% | 85.5% | 84.2% | 75.8% |
