# Supplementary material for: A novel member of the let-7 microRNA family is associated with developmental transitions in filarial nematode parasites
Source: BMC Genomics. 2015 Apr 22;16(1):331. doi: 10.1186/s12864-015-1536-y (PMC4428239; doi:10.1186/s12864-015-1536-y)
Supplement: Additional file 7: — Predicted miR-5364 target sites in orthologs of Bm1_ 27305 & Bm1_05425. [file 12864_2015_1536_MOESM7_ESM.docx]

**Predicted *mir-5364* target sites in orthologs of Bm1_ 27305 and Bm1_05425.**

The orthologs of Bm1_27305, Bm1_05425 and Bm1_25620 in the clade III parasitic nematodes *L. loa*, *W. bancrofti* and *L. sigmodontis* were examined for the presence of *miR-5364* target sites. *miR-5364* sites were identified in the orthologs of Bm1_27305 (Table A) and Bm1_05425 (Table B) from these three species in similar positions to those found for *B. malayi* and *D. immitis*. For Bm1_25620 although potential target sites were identified downstream of the orthologous genes in *W. bancrofti* and *L. sigmodontis*, site positioning was not well conserved, and target site prediction was not possible downstream of the *L. loa* ortholog due to a gap in the genome assembly (results not shown).

**A. Bm1_ 27305**

| **Species** | **Locus tag** | **Accession** | **Genomic contig ID** | **Annotated coding sequence end ^(a)^** | **PITA start co-ords ^(b)^** | **PITA ΔΔG score** |
| --- | --- | --- | --- | --- | --- | --- |
| *B. malayi* | Bm1_27305 | EDP34217.1 | DS239347 | 22304 | 93 | -10.39 |
| *D. immitis* | nDi.2.2.2.g03657 | NA | nDi.2.2.scaf00059 | 195810 | 94 | -7.6 |
| *L. loa* | LOAG_16420 | EJD76656 | JH712068.1 | 98640 | 101 | -8.84 |
| *W. bancrofti* | WUBG_02954 | EJW86133 | ADBV01000856.1 | 4333 | 98 | -9.85 |
| *L. sigmodontis* | - | - | 336655 | 3473 | 98 | -9.42 |

**B. Bm1_05425**

| **Species** | **Locus tag** | **Accession** | **Genomic contig ID** | **Annotated coding sequence end ^(a)^** | **PITA start co-ords ^(b)^** | **PITA ΔΔG score** |
| --- | --- | --- | --- | --- | --- | --- |
| *B. malayi* | Bm1_05425 | EDP38607.1 | DS237620 | 12739 | 36 | -9.67 |
| *D. immitis* | nDi.2.2.2.g05278 | NA | nDi.2.2.scaf00133 | 28443 | 47 | -11.91 |
| *L. loa* | LOAG_06170 | EFO22316 | JH712204.1 | 36534 | 47 | -11.05 |
| *W. bancrofti* | WUBG_03396 | EJW85695 | ADBV01001033.1 | 11486 | 35 ^(c)^ | -11.08 |
| *L. sigmodontis* | - | - | 344523 | 2531 | 48 | -13.14 |

^(a)^ For *L. sigmodontis* the stop codon was determined from the BLAST result at 959 Nematode Genomes.

^(b)^ Number of nucleotides downstream of annotated coding sequence that the *mir-5364* site is predicted to start.

^(c)^ For WUBG_03396 the sequence downstream from the annotated stop was compared with sequence from other *W. bancrofti* contigs at 959 Nematode Genomes where contig ADBV01002892.1 indicated an additional nucleotide compared to ADBV01001033.1, and it was this sequence (TGCAAAAGTAATGACATGCTTCAATATATAATTGATACCTCTTATTTTGTACTCAAAAATATTCTCAATGATTATCAAAGTGTTAA, additional nucleotide underlined) that was used for the PITA analysis.
