# Supplementary figures and images for: A novel member of the let-7 microRNA family is associated with developmental transitions in filarial nematode parasites
Source: BMC Genomics. 2015 Apr 22;16(1):331. doi: 10.1186/s12864-015-1536-y (PMC4428239; doi:10.1186/s12864-015-1536-y)

**A B**


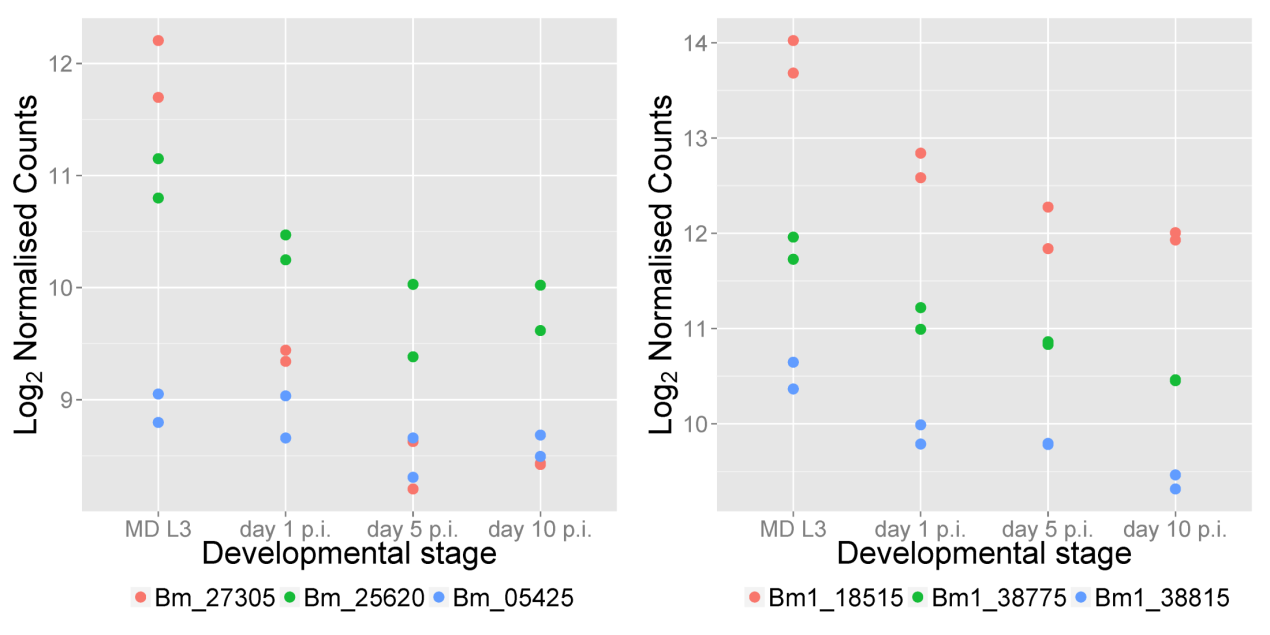

Supplement: Additional file 8: — Expression profiles of predicted bpa-miR-5364 targets by RNAseq. [file 12864_2015_1536_MOESM8_ESM.docx]
