# Supplementary material for: A novel member of the let-7 microRNA family is associated with developmental transitions in filarial nematode parasites
Source: BMC Genomics. 2015 Apr 22;16(1):331. doi: 10.1186/s12864-015-1536-y (PMC4428239; doi:10.1186/s12864-015-1536-y)
Supplement: Additional file 10: — Oligonucleotide primer and synthetic gene sequences. [file 12864_2015_1536_MOESM10_ESM.docx]

**Oligonucleotide primer and synthetic gene sequences.**

*B. pahangi* material was used for PCR in all cases. Therefore, in this work, protein coding genes with identifiers of the type “Bm1_00001” refer to the *B. pahangi* ortholog of the *B. malayi* gene.

| **Name** | **Sequence, 5′-3′** | **Use** |
| --- | --- | --- |
| oADW082 | CGAGGTATTGTTTATTGGCT (20) | *bpa-mir-5364* qRT-PCR forward primer. |
| oADW092 | AACCCGTAGAACTGAAATC (19) | *bpa-mir-100c* qRT-PCR forward primer, normalising miRNA. |
| oADW0140 | **ucaCA**UCAGCCAAUAAACAAUACCUCG**ACcuu** (32) | Antisense oligo to inhibit *bpa-mir-5364*. All 2'O-methyl RNA. The bases in bold are added and do not correspond to the miRNA sequence. Bases in lower case are joined by phosphorothioate bonds. |
| oADW0191 | **ucaCA**UUAAUCGCGUCCAACACAACAA**ACcuu** (32) | Scrambled *bpa-mir-5364* antisense oligo. All 2'O-methyl RNA. Bases in lower case are joined by phosphorothioate bonds. |
| Bm1_27305 3′RACE 1 | GCAGAAGGCGTTTTTCGAATTG (22) | Bm1_27305 (Ets-domain containing protein) 3′RACE primer 1. |
| Bm1_27305 3′RACE 2 | GGCACAACTTTGGGGTGAAC (20) | Bm1_27305 (Ets-domain containing protein) 3′RACE primer 2. |
| Bm1_05425 3′RACE 1 | GACTGATCTCACAGATTGTATGC (23) | Bm1_05425 (Zinc finger in N-recognin family protein) 3′RACE primer 1. |
| Bm1_05425 3′RACE 2 | GATGTGGCTGGTCGTGAAG (19) | Bm1_05425 (Zinc finger in N-recognin family protein) 3′RACE primer 2. |
| Bm1_25620 3′RACE 1 | GATCCGAATGCACCGAAG (18) | Bm1_25620 (high mobility group protein) 3′RACE primer 1. |
| Bm1_25620 3′RACE 2 | GCATGTCTGTCATTGATGTGAG (22) | Bm1_25620 (high mobility group protein) 3′RACE primer 2. |
| Bm1_27305 F (Q) | CTGCATCAGTAGTGGACTGTC (21) | Forward primer for Bm1_27305 (Ets-domain containing protein) qRT-PCR. |
| Bm1_27305 R (Q) | CTTTGCAATATGTCGGTCGTAAC (23) | Reverse primer for Bm1_27305 (Ets-domain containing protein) qRT-PCR. |
| Bm1_ 05425 F (Q) | CGACACCGAACAAGAGGATATC (22) | Forward primer for Bm1_05425 (Zinc finger in N-recognin family protein) qRT-PCR. |
| Bm1_ 05425 R (Q) | GTGCTTCGATAACTTCACTTAATG (24) | Reverse primer for Bm1_05425 (Zinc finger in N-recognin family protein) qRT-PCR. |
| Bm1_25620 F (a) (Q) | GTCAAGTAAAAGGTCAAGC (19) | Forward primer for Bm1_25620 (high mobility group protein) qRT-PCR. |
| Bm1_25620 R (a) (Q) | GCCCCATTCAACACCTGCTGC (21) | Reverse primer for Bm1_25620 (high mobility group protein) qRT-PCR. |
| Brugia btub F (Q) | GCTGGTCAATGTGGCAACCAG (21) | Forward primer for beta-tubulin (AY705382) qRT-PCR, normalising mRNA. |
| Brugia btub R (Q) | GCAAGTCTGAATCACCTTTGTATG (24) | Reverse primer for beta-tubulin (AY705382) qRT-PCR, normalising mRNA. |
| Bm1_27305For (3′UTR) | GCGGCCGCTGTATCCGAAGAAGTTAGTG (28) | Forward primer for Bm1_27305 (Ets-domain containing protein) 3′UTR. Not I site underlined. |
| Bm1_27305Rev (3′UTR) | GCGGCCGCTAGTTCCGTCTCGACATTTTC (29) | Reverse primer for Bm1_27305 (Ets-domain containing protein) 3′UTR. Not I site underlined. |
| oADW0179 | AAGCGGCCGCgaacttgaaatggaacgtaaac (32) | Forward primer for Bm1_05425 (Zinc finger in N-recognin family protein) 3′UTR. Not I site underlined. |
| oADW0180 | AAGCGGCCGCtaactgtacctcaacatatag (31) | Reverse primer for Bm1_05425 (Zinc finger in N-recognin family protein) 3′UTR. Not I site underlined. |
| oADW0200 | GGCGGCCGCCAAGAAGTAACAGTGAAGAAG | Forward primer for Bm1_25620 (high mobility group protein) 3′UTR. Not I site underlined. |
| oADW0182 | AAGCGGCCGCtagcctgatcgatattacaac (31) | Reverse primer for Bm1_25620 (high mobility group protein) 3′UTR. Not I site underlined. |
| oADW0183 | GAAGGTACCagcttcacttttcggtaatattcg (33) | Forward primer for *bpa-mir-5364*. Kpn I site underlined. |
| oADW0184 | AAGCGGCCGCCAACATCCACGAAGAAGTATGAAA (34) | Reverse primer for *bpa-mir-5364*. Not I site underlined. |
| Synthetic gene, 5X bpa-mir-5364 perfect target | GAATTCTCAGCCAATAAACAATACCTCGatagctTCAGCCAATAAACAATACCTCGaccaacTCAGCCAATAAACAATACCTCGtcgagcTCAGCCAATAAACAATACCTCGacgtacTCAGCCAATAAACAATACCTCGGCGGCCGC | 5X reverse complement of *bpa-mir-5364* with each target site separated by 6 nucleotides of random sequence (shown in lower case). Eco RI and Not I sites are underlined. |
| oADW072 | ATGGCACCATATCAACCCTTTC (22) | Forward primer for full length predicted sequence of Bm1_13960 (argonaute 2) mRNA. |
| oADW073 | TCAAGCGAAATACATGACATTG (22) | Reverse primer for full length predicted sequence of Bm1_13960 (argonaute 2) mRNA. |
